# Radiosensitization and downregulation of heterogeneous nuclear ribonucleoprotein K (hnRNP K) upon inhibition of mitogen/extracellular signal-regulated kinase (MEK) in malignant melanoma cells

**DOI:** 10.18632/oncotarget.3935

**Published:** 2015-05-22

**Authors:** Stefan Eder, Andreas Lamkowski, Markus Priller, Matthias Port, Konrad Steinestel

**Affiliations:** ^1^ Bundeswehr Institute of Radiobiology, Neuherbergstrasse 11, 80937 Munich, Germany; ^2^ Gerhard-Domagk-Institute of Pathology, University Hospital Muenster, Domagkstrasse 17, 48149 Muenster, Germany

**Keywords:** nRNP K, MEK inhibition, NRAS, radiotherapy, melanoma

## Abstract

**Background:**

Heterogeneous nuclear ribonucleoprotein K (hnRNP K) is an important cofactor in the p53-mediated DNA damage response pathway upon ionizing radiation (IR) and exerts anti-apoptotic effects also independent of p53 pathway activation. Furthermore, hnRNP K is overexpressed in various neoplasms including malignant melanoma (MM). Here, we investigate the role of hnRNP K in the radioresistance of MM cells.

**Methods and results:**

Our results show cytoplasmic expression of hnRNP K in human MM surgical specimens, but not in benign nevi, and a quick dose- and time-dependent upregulation in response to IR accompanied by cytoplasmic redistribution of the protein in the IPC-298 cellular tumor model carrying an activating NRAS mutation (p.Q61L). SiRNA-based knockdown of hnRNP K induced a delayed decline in γH2AX/53BP1-positive DNA repair foci upon IR. Pharmacological interference with MAPK signaling abrogated ERK phosphorylation, diminished cellular hnRNP K levels, impaired γH2AX/53BP1-foci repair and proliferative capability and increased apoptosis comparable to the observed hnRNP K knockdown phenotype in IPC-298 cells.

**Conclusion:**

Our results indicate that pharmacological interference with MAPK signaling increases vulnerability of *NRAS*-mutant malignant melanoma cells to ionizing radiation along with downregulation of endogenous hnRNP K and point towards a possible use for combined MEK inhibition and localized radiation therapy of MM in the *NRAS*-mutant setting where BRAF inhibitors offer no clinical benefit.

## BACKGROUND

Malignant melanoma (MM) is commonly regarded as a radioresistant tumor entity, although adjuvant radiotherapy plays a role in treatment regimens for patients suffering from advanced MM by reducing the risk of local and metastatic tumor relapse [1, 2]. Several studies, including a randomized phase III multicenter clinical trial, showed that postoperative radiation therapy significantly reduces the risk of lymph node recurrence in patients who have undergone therapeutic lymphadenectomy, yet leaving overall survival dissatisfyingly unchanged [3, 4]. A better understanding of the mechanisms that enable melanoma cells to resist to ionizing radiation (IR)-induced DNA damage might thus improve strategies for melanoma management.

Shortly after DNA damage, the cellular p53 caretaker system initiates cell cycle arrest and expression of proteins involved in DNA damage response (DDR). Previously, the presence of heterogeneous nuclear ribonucleoprotein K (hnRNP K) has been described to be essential for p53-mediated effects following DNA damage [5]. HnRNP K is an ubiquitously expressed ribonucleoprotein capable of shuttling between the nucleus, cytoplasm and mitochondria [6]. Three K-homology (KH) domains enable hnRNP K to bind specifically to poly(C)-rich DNA and pre-mRNA sequences; the protein is thus closely involved in regulating gene expression and posttranscriptional mRNA modifications, including mRNA maturation and translation. The pivotal functions of hnRNP K for cell survival and homeostasis are regulated by a fine adjustment of its subcellular (nuclear or cytoplasmic) localization, phosphorylation state and protein-protein interactions [7–9]. The ataxia-telangiectasia-mutated (ATM) protein kinase directly phosphorylates hnRNP K, facilitating its stabilization and cytoplasmic accumulation by inhibiting E3 ubiquitin-protein ligase, Mdm2-mediated ubiquitination and subsequent proteasomal degradation. A multi-protein complex containing hnRNP K and p53 then binds to p53 promoter sites, resulting in the activation of DDR gene expression [5, 10]. Alternatively, cytoplasmic accumulation of the protein has been shown to stabilize the mRNA for Thymidine phosphorylase, a major inhibitor of apoptosis [9]. In summary, hnRNP K is a key player in the cellular response to DNA damage and inhibition of apoptosis.

There is growing evidence that hnRNP K is also implicated in tumorigenesis and the gain of a metastatic phenotype of malignant tumors by deregulating the transcription and/or translation of multiple cellular oncogenes [11, 12]. Since the protein has been shown to be highly expressed in various cancers, increasing efforts have been made to characterize its possible value as a biomarker [13–15]. A recent study revealed high expression of hnRNP K in MM tissue specimens and cell lines; knockdown of the protein impaired MM-tumor growth and colony formation in that study [16]. In accordance to its pivotal role as a transcription factor and post-transcriptional mRNA modifying protein, hnRNP K is a target for multiple intracellular signaling pathways, including the mitogen-activated protein kinase (MAPK) pathway [8, 17, 18]. Activating mutations in MAPK signaling proteins are frequent in MM. A recent meta-analysis of 4493 patients with primary cutaneous melanoma showed *BRAF*/*NRAS* mutations in 41% and 18%, respectively [19]. Thus, enhanced phosphorylation of extracellular-signal-regulated kinase (ERK) is frequently observed in MM downstream *NRAS* and *BRAF* and, importantly, induces cytoplasmic accumulation of hnRNP K [18, 20]. Taken together, experimental evidence suggests a possible role for the MAPK-hnRNP K-DDR axis in the radioresistance of MM cells. In this study, we analyzed hnRNP K expression patterns and subcellular localization in benign and MM tissue applying immunohistochemistry. To investigate the significance of MAPK-mediated upregulation of hnRNP K in radioresistance of MM, we analyzed the effects of IR in a MM cellular tumor model carrying an activating *NRAS* p.Q61L mutation (IPC-298). Finally, we examined the impact of hnRNP K on the DNA damage response pathway using siRNA knockdown of hnRNP K as well as the mitogen/extracellular signal-regulated kinase (MEK) inhibitor PD98059.

## RESULTS

### HnRNP K expression in tissue specimens

**Figure 1:**
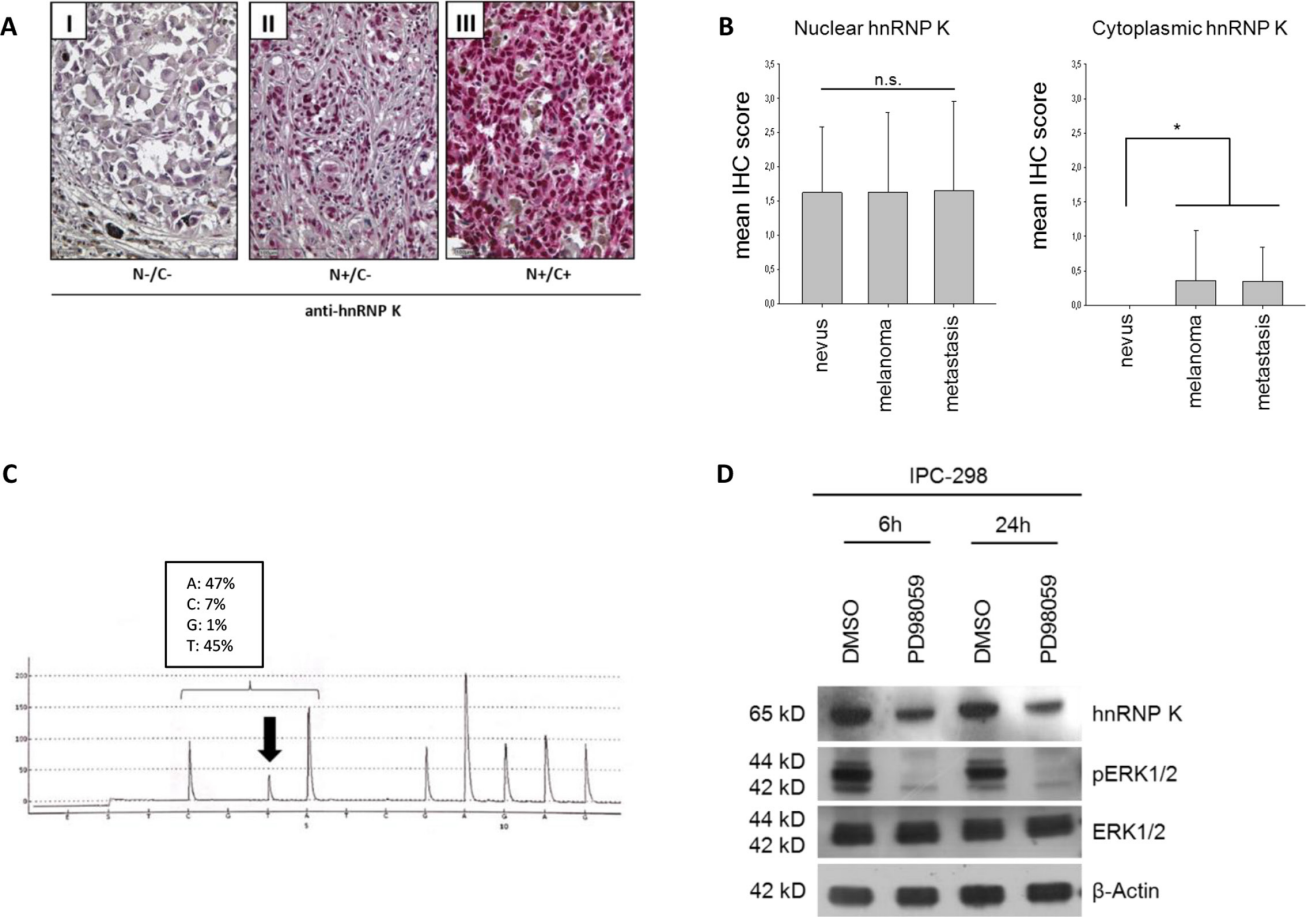
**A. Expression of hnRNP K in malignant melanoma tissue specimens.** Criteria for classifying nuclei (N) and cytoplasm (C) as positive or negative for hnRNP K are illustrated by representative immunohistochemistry for hnRNP K. **B.** IHC scoring was done by separately analyzing hnRNP K staining intensity for nuclear and cytoplasmic hnRNP K expression. Mean nuclear IHC scores of benign nevi, malignant melanoma (MM) or MM metastasis showed no significant difference. 25% of MM and MM metastases stained positively for cytoplasmic hnRNP K whereas nevi were completely devoid of cytoplasmic hnRNP K expression (n: nevi = 18, melanoma = 62, metastasis = 20; *p < 0.05 versus nevi). **C.** Verification of *NRAS* mutation in IPC-298 cells by DNA pyrosequencing. The CAA to CTA transition leads to a p.Q61L amino acid substitution, comprising constitutive signaling activity along the MAPK pathway. **D.** Inhibition of MEK signaling by 50 μM PD98059 abrogates phosphorylation of ERK1/2 and reduces endogenous hnRNP K levels.

To analyze hnRNP K expression patterns in MM *in vivo*, we performed immunohistochemistry on a human melanoma tissue microarray containing 18 benign nevi, 62 malignant melanomas and 20 MM metastases; detailed clinico-pathological sample characteristics are summarized in Table 1. For IHC scoring, we separately evaluated hnRNP K expression in the nuclei and the cytoplasm of tumor cells (Fig. 1A). HnRNP K immunopositivity was detected in 75% of MM and 70% of MM metastasis specimens, respectively. There was no significant difference in nuclear hnRNP K staining between diagnostic groups (nevi/MM/metastases); in contrast, cytoplasmic hnRNP K positivity was only detected in MM and MM metastases (25% of cases), but not in benign nevi (Fig. 1B).

**Table 1:** Clinic-pathologic sample characteristics

No. of patients	100
**No. of samples**	**100 (100%)**
**Median age (years)**	51 ± 17.9
**Gender** Male Female	55 (55%) 45 (45%)
**Sample location** Skin Head/Neck Rump Extremities Metastases Lymph node Soft tissue Other	80 (80%) 14 (18%) 43 (53%) 23 (29%) 20 (20%) 11 (55%) 8 (40%) 1 (5%)
**Histology** Melanocytic Nevus Sebaceous Nevus Malignant Melanoma (MM) Metastatic MM	16 (16%) 2 (2%) 62 (62%) 20 (20%)
**UICC Stage (*n* = 58)** I II III IV	6 (10%) 26 (45%) 24 (41%) 2 (4%)

### Elevated endogenous hnRNP K levels in *NRAS*-mutant IPC-298 melanoma cells

IPC-298 MM cells carry an activating *NRAS* mutation (Q61L) [21]. To reconfirm this, we performed DNA pyrosequencing after DNA isolation and verified a heterozygous CAA to CTA transition in codon 61 of the *NRAS* gene, leading to a p.Q61L amino acid substitution (Fig. 1C). After incubation of IPC-298 cells with 50 μM of PD98059 for 6 h and 24 h, phospho-ERK levels were barely detectable and parallel to the decline in ERK phosphorylation, there was a decrease in endogenous hnRNP K levels after 6 h of incubation with subsequent stabilization at a lower expression level for up to 24 h (Fig. 1D). These results indicate a positive correlation between MAPK signaling activity and hnRNP K expression levels in *NRAS*-mutant IPC-298 cells.

### HnRNP K protein expression in response to IR

**Figure 2:**
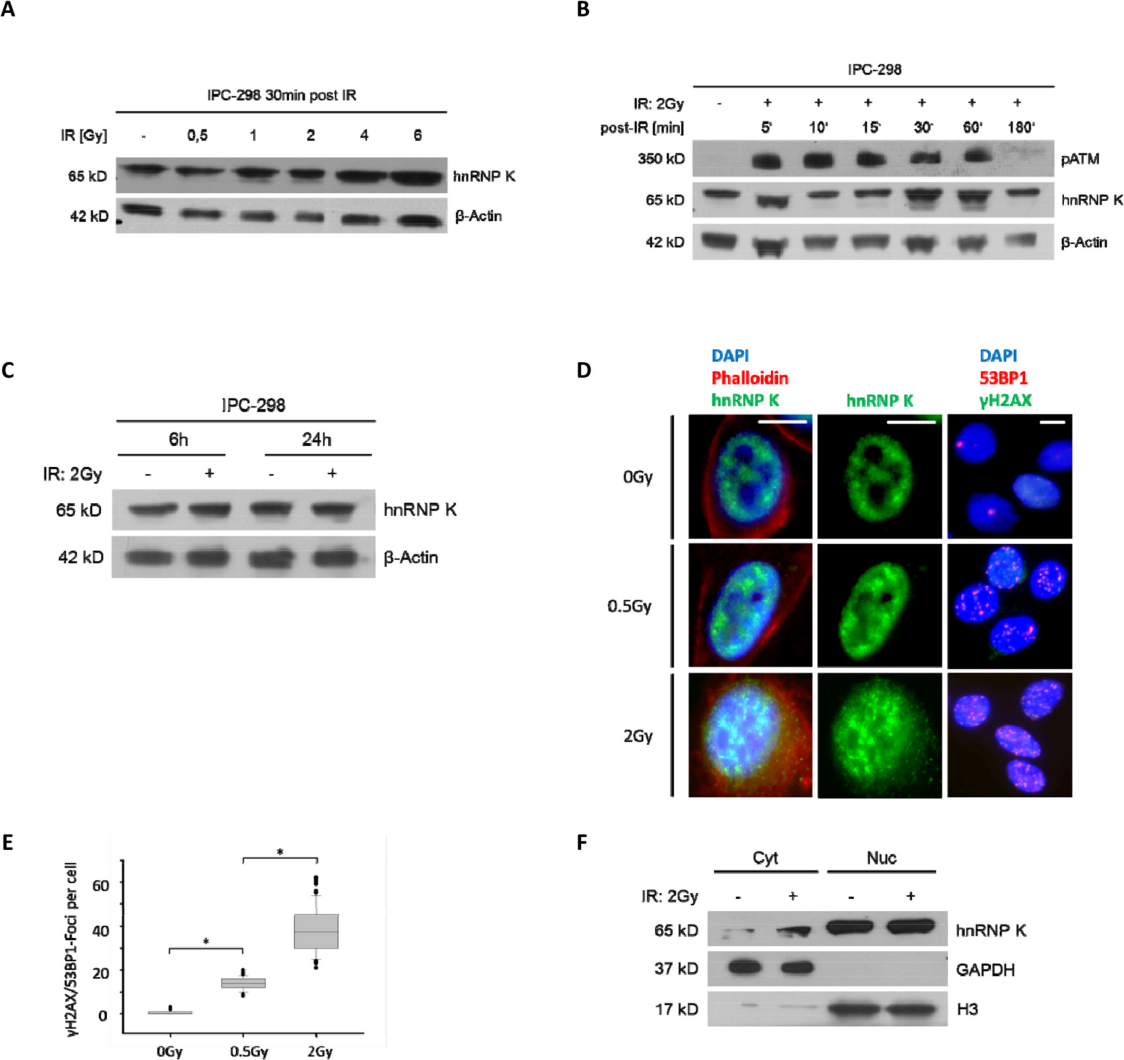
**A. Immunoblotting of hnRNP K shows dose-dependent upregulation within 30 minutes after IR. B.** Immunoblotting of IPC-298 cell lysate showing time-dependent increase in intensity of the hnRNP K-double band with a maximum at 30 – 60 minutes and a subsequent normalization after 180 minutes, parallel to the detection of phospho-ATM. **C.** 6 hours and 24 hours after applying IR (2 Gy), there is no difference in hnRNP K expression between irradiated and non-irradiated samples. **D.** Immunofluorescence microscopy of IPC-298 cells shows strict nuclear localization of hnRNP K under control conditions. Within 30 minutes, exposure to IR causes perinuclear accumulation of hnRNP K in a dose-dependent manner. Scale bars represent 30μm. **E.** DNA damage repair as illustrated by dose-dependent increase in γH2AX/53BP1-Foci (*n* = 100; *p* < 0.05). **F.** Subcellular fractionation and immunoblotting reveals cytoplasmic (Cyt) accumulation of hnRNP K 30 min post-IR (2 Gy). Nuclear (Nuc) hnRNP K levels remain unchanged.

To investigate the effects of ionizing radiation on hnRNP K protein expression, IPC-298 cells were X-rayed with doses reaching from 0.5 to 6 Gy (Fig. 2A). HnRNP K accumulated in a dose-dependent manner from 0.5 to 6 Gy with an additional band appearing upon 0.5 Gy and increasing signal strength upon higher IR doses. Additionally, analyses of time-dependent changes in protein expression were performed after 2 Gy-irradiation, representing the commonly delivered daily dose in most standard fractionation regimes in radiotherapy. Cellular hnRNP K levels rapidly increased after 2 Gy-irradiation, ascending to a plateau between 30min and 60 min and re-descending to baseline after 3 h. Radiation-induced phosphorylation of ATM could be observed simultaneously (Fig. 2B). HnRNP K levels remained unaltered 6 and 24 hours following irradiation (Fig. 2C).

### Ionizing radiation leads to cytoplasmic accumulation of hnRNP K

To specify the subcellular localization patterns of hnRNP K upon IR, we performed immunofluorescence stainings of IPC-298 cells. Under control conditions, hnRNP K followed a strictly nuclear localization pattern in IPC-298 cells (Fig. 2D, upper row). 30min after 0.5 or 2 Gy-irradiation, respectively, we observed a dose-dependent perinuclear accumulation of hnRNP K as well as a significant increase in γH2AX/53BP1-positive DNA damage repair foci (Fig. 2D and 2E). To verify this observation, we performed western immunoblotting of cytoplasmic and nuclear cellular fractions and found cytoplasmic hnRNP K levels to be elevated 30 min after 2 Gy (Fig. 2F). These findings indicate that the overall increase in hnRNP K expression upon IR is due to a cytoplasmic rather than nuclear accumulation of the protein.

### Knockdown of hnRNP K impairs DNA damage response and clonogenic viability in MM

**Figure 3:**
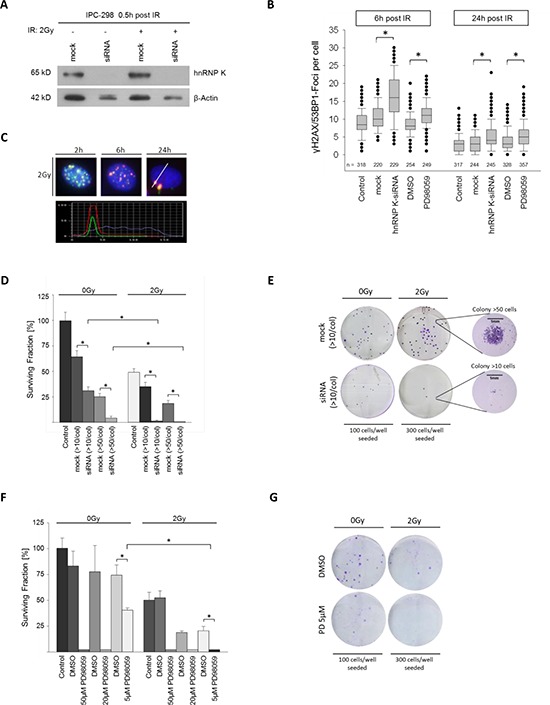
**A. Immunoblotting shows effective knockdown of hnRNP K. B.** Knockdown of hnRNP K and MEK inhibition result in a significantly reduced decline in γH2AX/53BP1 foci (*p < 0.05). **C.** Time-dependent DNA-damage response (DDR) shown by γH2AX/53BP1 foci upon 2 Gy IR. Co-localization of γH2AX/53BP1 was verified by staining intensity profile. **D.** Knockdown of hnRNP K evokes radiosensitization of IPC-298 cells (*n* = 4). **F.** MEK inhibition with PD98059 impairs clonogenic viability of IPC-298 melanoma cells in a dose-dependent manner (*n* = 4). **G.** Representative colony formation assays show partial depletion of clonogenic cell survival by treatment with 5 μM PD98059 and complete abrogation by additional irradiation with 2 Gy (*n* = 4).

To analyze the role of hnRNP K in DDR, we knocked down hnRNP K in IPC-298 cells using siRNA; mock transfections with nonsense siRNA were performed as control experiments. Transfection with hnRNP K siRNA almost completely abrogated cellular hnRNP K levels while in contrast, mock siRNA transfection showed no comparable effect (Fig. 3A). 30 min after 2 Gy-irradiation, hnRNP K protein levels remained undetectable in western immunoblotting. However, levels of β-Actin were also diminished to some extent after knockdown of hnRNP K despite equal protein loading in all gel slots (Fig. 3A). To investigate the DDR in the absence of hnRNP K, we repeated the immunofluorescence staining for γH2AX and 53BP1 and found the time-dependent decline of γH2AX/53BP1-Foci to be significantly delayed upon hnRNP K knockdown. This result was persistent until 24 h after IR and turned out statistically significant (Fig. 3B and 3C). Clonogenic cell survival assays showed that presence of the transfection reagent decelerated colony formation. Notably, siRNA-mediated depletion of endogenous hnRNP K reduced colony size (< 50 cells per colony) after incubation for 14 days. Finally, additional 2 Gy-irradiation completely abrogated clonogenic viability of IPC-298 MM cells (Fig. 3D and 3E). Taken together, these results indicate that hnRNP K plays an essential role in the DNA damage response and clonogenic viability of human malignant melanoma cells.

### MEK inhibition impairs γH2AX/53BP1 foci repair and colony formation mirroring the hnRNP K knockdown phenotype in IPC-298 cells

Since MAPK signaling activity had a strong impact on endogenous hnRNP K levels in IPC-298 cells (Fig. 1D), we analyzed the effects of MEK inhibition (MEKi) towards IR-induced DDR. Similar to hnRNP K knockdown, MEKi significantly impaired radiation-induced DSB-repair, as shown by γH2AX/53BP1 immunofluorescence staining (Fig. 3B). To assess cell survival and proliferation upon MEKi, we exposed IPC-298 cells to increasing concentrations of PD98059. While colony formation could still be observed upon application of 5 μM PD98059, 20 μM and 50 μM of the compound completely abrogated colony formation comparable to the hnRNP K siRNA knockdown phenotype. However, additional exposure to IR (2 Gy) abolished the observed resurgence in clonogenic viability (Fig. 3F and 3G). This suggests that interference with MAPK signaling radiosensitizes human malignant melanoma cells comparable to the hnRNP K siRNA knockdown phenotype.

### Downregulation of hnRNP K through MEK inhibition enhances the apoptotic fraction in IPC-298 cells upon IR

**Figure 4:**
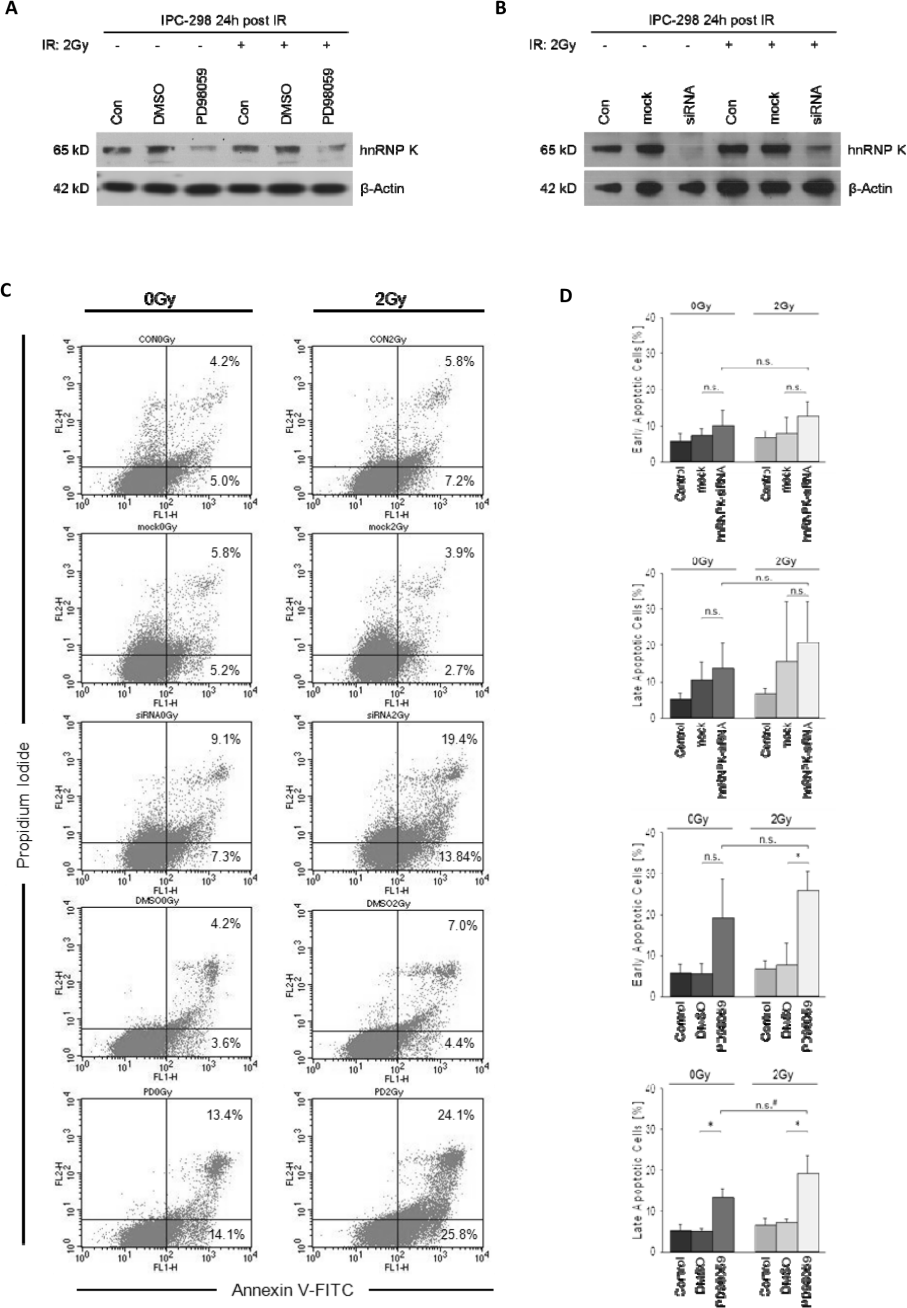
**A and B. MEK inhibition with PD98059 for 24 hours post-IR impairs normalization of cellular hnRNP K levels in irradiated and non-irradiated IPC-298 cells comparable to the hnRNP K knockdown phenotype. C.** Effects of MEK inhibition and hnRNP K knockdown on apoptosis were analysed by flow cytometry using Annexin V-FITC/PI staining. Presented data show the results of a contiguous test series. Early apoptosis is represented by Annexin V^+^/PI^−^ events, whereas Annexin V^+^/PI^+^ counts demonstrate late apoptosis/necrosis. **D.** Statistical analyses of flow cytometry results. Experiments were performed in triplicate. Untreated cells served as control. The presented columns are given as the means ± S.D. (*p < 0.05, n.s. = not significant, ^*^p = 0.05).

Since both MEK inhibition and hnRNP K knockdown have been shown to independently mediate apoptotic effects, we tested whether this is also true for the downregulation of endogenous hnRNP K through inhibition of MAPK-signalling. Therefore, cells were either preincubated with DMSO/PD98059 or pretransfected with mock/hnRNP K siRNA, respectively. After 2 Gy-irradiation, cells were allowed to recover for 24 h. Both treatments effectively diminished hnRNP K levels irrespective of whether cells were previously irradiated (Fig. 4A and 4B). Contrary to the absence of cellular hnRNP K in siRNA-transfected cells 0.5 h post-IR (Fig. 3A), 24 h of recovery led to reappearance of a band at the correct molecular weight (65 kD), yet with greatly reduced intensity compared to mock transfection (Fig. 4B). Flow cytometry analyses revealed that 2 Gy-irradiation alone had no effect on the rate of early or late phase apoptosis in IPC-298; in contrast, MEK inhibition significantly increased the percentages of both early and late apoptotic/necrotic cells upon IR as well as the percentage of late apoptotic cells under control conditions (Fig. 4C and 4D, *p* < 0.05). HnRNP K siRNA knockdown also induced apoptosis, yet the difference to mock transfection was not significant. Both hnRNP K siRNA knockdown and MEK inhibition led to the highest fraction of early and late apoptosis under irradiated compared to control conditions, respectively; this result, however, failed to reach statistical significance (*p* = 0.05).

## DISCUSSION

In the present study, we analyzed the impact of MEK inhibition on DNA damage response and cell survival upon ionizing radiation in *NRAS*-mutant malignant melanoma cells and show first evidence for the hypothesis that a functional cellular DDR in MM requires MAPK-mediated upregulation of hnRNP K.

High expression levels of hnRNP K have been observed in many cancers, including MM, and correlate with tumor initiation, tumor progression and poor prognosis [14–16, 22, 23]. Applying immunohistochemistry, we found that hnRNP K follows a nuclear expression pattern in benign nevi, MM and MM metastases, while cytoplasmic immunoreactivity is only observed in a subset of MM and MM metastases, but not in nevi. These results support previously reported findings [16], however, the cytoplasmic redistribution of hnRNP K has so far not been linked to the radioresistance of MM cells.

To assess the role of hnRNP K in the DNA damage response with respect to MAPK signaling activity in MM, we used *NRAS*-mutant IPC-298 MM cells as an *in vitro* tumor model. We could confirm a previously described p.Q61L mutation of the *NRAS* gene in IPC-298 cells, leading to constitutive activation of cellular MAPK signaling [21]. Irradiated cells showed quick upregulation of hnRNP K expression in a dose- and time-dependent manner. Recently, phosphorylation of hnRNP K as a prerequisite for evading degradation through MDM- 2-mediated ubiquitination has been shown to be an ATM-dependent process [5, 10]. This is in line with the observed parallel increase in both cellular hnRNP K and phospho-ATM levels upon IR that we observed. Appearance of an additional band with smaller molecular weight upon IR might thus correspond to the non-ubiquitinated isoform of hnRNP K in the presence of phospho-ATM.

Given its multiple nuclear and cytoplasmic functions, hnRNP K is capable of leaving the nucleus and shuttling to the cytoplasm and to mitochondria, respectively [8, 17]. Both immunofluorescence and immunoblotting experiments revealed strict nuclear localization of hnRNP K in IPC-298 cells under control conditions and a rapid cytoplasmic and perinuclear accumulation of the protein subsequent to IR. This correlated to the extent of DNA damage as shown by γH2AX/53PB1 focus analysis; the number of repair foci has been previously shown to correlate with both the absorbed IR dose and the extent of DNA damage [24–28]. Abrogation of endogenous hnRNP K levels using a siRNA-based approach significantly increased persistence of radiation-induced γH2AX/53PB1-foci in addition to a complete loss of proliferative ability in IPC-298 cells as shown by clonogenic survival assay. These findings underline the pivotal role of hnRNP K in the radioresistance of human MM cells.

To date, the association between MAPK signaling and hnRNP K expression remains controversial. While a positive correlation was found in HeLa and leukemia cells, a previous study showed no significant correlation between levels of phospho-ERK and hnRNP K in MM tissue specimens and cell lines, respectively [16, 18, 29]. In IPC-298 melanoma cells, displaying constitutively activated MAPK-signaling upon an activating *NRAS* p.Q61L mutation, MEK inhibition significantly decreased cellular hnRNP K levels. To test for the functional consequences of this observation, cells were pretreated with MEK inhibitor (PD98059) prior to IR exposure. Identical to the hnRNP K knockdown phenotype, MEK-inhibition resulted in increased persistence of γH2AX/53PB1 foci and abrogation of colony formation. While some degree of clonogenic survival of MM cells was sustained upon low concentrations of PD98059 (5 μM), this was abrogated by exposure to 2 Gy-irradiation. The observed radiosensitizing effect of MEK-inhibition is in line with findings from other authors [30].

**Figure 5:**
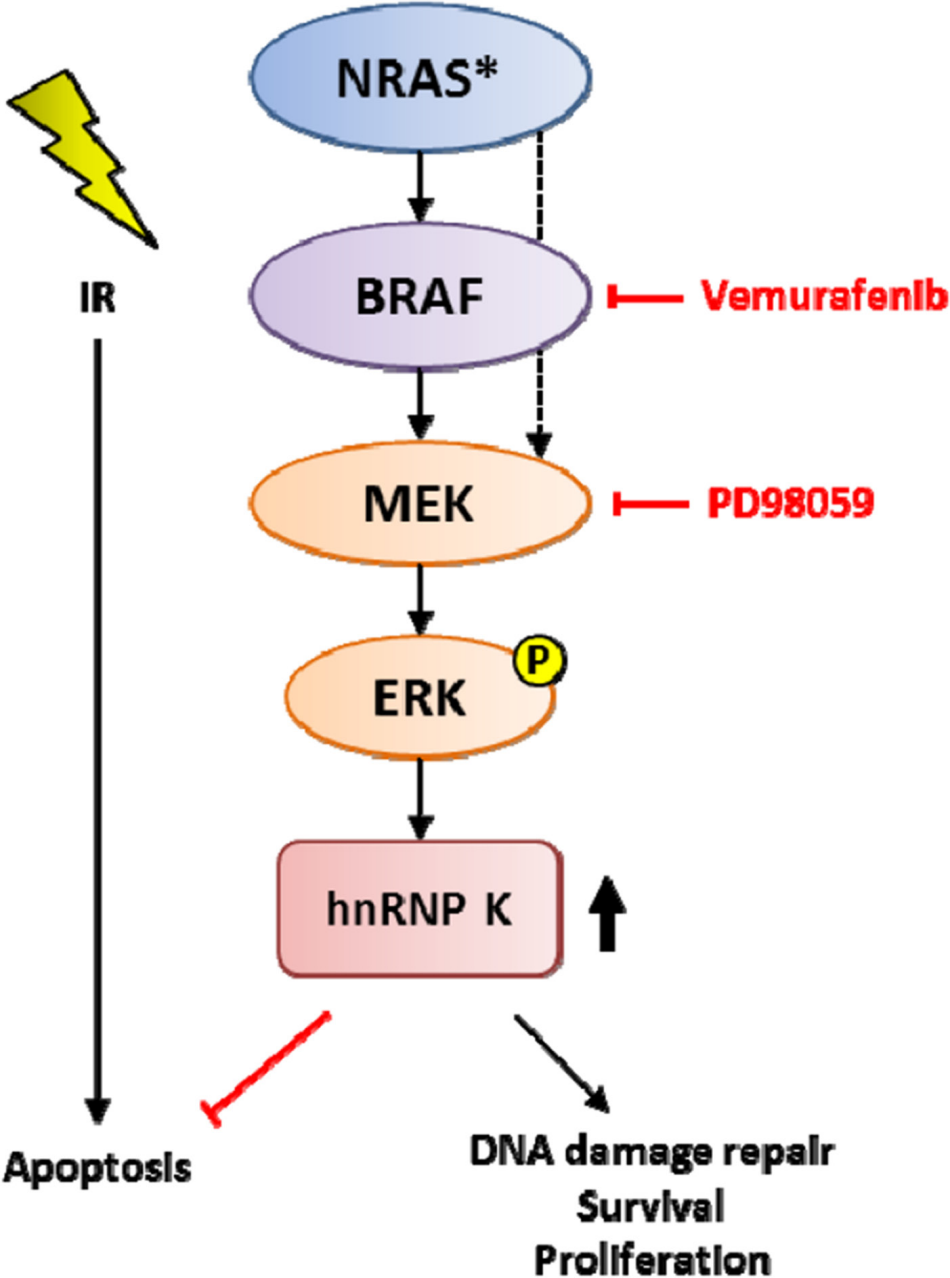
Schematic model for the role of MAPK-mediated upregulation of hnRNP K in the DDR pathway and possible targets for pharmacological interference.

Furthermore, both MEK inhibition and hnRNP K knockdown have been previously linked to enhanced apoptosis [31–36]. Here, we could show that both inhibition of MAPK signaling and hnRNP K siRNA knockdown not only exert pro-apoptotic effects as single treatments, but that interference with MAPK signaling enhances the apoptotic effect of ionizing radiation on MM cells mirroring the hnRNP K knockdown phenotype. The results for hnRNP K knockdown in flow cytometry analyses, however, just failed to reach statistical significance and thus, have to be interpreted with caution; possible explanations are the toxic effects of liposomal cell transfection (that could also be observed in the clonogenic viability assays) and the impact of MEK inhibition on other pro-apoptotic pathways unrelated to hnRNP K. Nevertheless, since the basic findings are in line with the results from our DNA damage response and clonogenic viability assays and since we show downregulation of hnRNP K in response to MEK inhibition, we conclude that the upregulation and cytoplasmic accumulation of hnRNP K is highly dependent on MAPK signaling activity and mediates radioprotective effects in MM; a possible model comprising our main findings is depicted in Fig. 5. Taken together, the high baseline radioresistance of melanocytic malignancies might be a consequence of the observed high cytoplasmic hnRNP K levels in these lesions, possibly due to hnRNP K-mediated inhibition of caspase activity as has been previously reported for hepatocellular carcinoma cells under treatment with 5-fluorouracil (5-FU) [34]. Moreover, MAPK-driven cytoplasmic accumulation and phosphorylation of hnRNP K induces expression of thymidine phosphorylase (platelet-derived endothelial cell growth factor, PD-ECGF/TP), an inhibitor of caspase 3 and 9 activation and mitochondrial cytochrome c release, in nasopharyngeal carcinoma cells [9, 37]. It would thus be of great interest to analyze expression levels of PD-ECGF/TP and the impact on caspase activation and apoptosis in irradiated MM cells in further studies.

Our findings imply that MEK inhibition might enhance vulnerability of *NRAS* mutant melanoma cells to DNA damage induced not only by radiation, but also by chemotherapy. This is of clinical relevance, since activating *BRAF* mutations are frequently observed in MM but the therapeutic benefit of selective BRAF inhibitors like vemurafenib may be limited due to bypassing mechanisms like secondary *NRAS* mutations [38]. Drugging MEK as a downstream target of activated MAPK signaling might therefore be a promising therapeutic approach for the treatment of vemurafenib-resistant tumours [20, 39, 40]; the results presented here suggest a possible additional use of MEK inhibition through the impairment of DDR and enhanced apoptosis, possibly via downregulation of hnRNP K.

## CONCLUSIONS

In this study, we demonstrate high levels of cytoplasmic hnRNP K in tissue samples of malignant melanoma (MM) and MM metastases compared to benign nevi; moreover, we show for the first time that presence of hnRNP K is essential for DNA damage repair upon IR in *NRAS*-mutant MM cells. This might be dependent on MAPK signaling activity as MEK inhibition prior to ionizing radiation downregulated hnRNP K, impaired DNA damage repair, cell survival and proliferative capability and enhanced apoptosis in MM cells. Since *NRAS* mutations are found in 18% of MM and are furthermore observed as an escape mechanism against first-line vemurafenib treatment in *BRAF*-mutant MM, MEK-inhibition could represent a promising therapeutic target to enhance the susceptibility of melanoma cells to therapy-induced DNA damage.

## MATERIALS AND METHODS

### Tissue microarray and immunohistochemical (IHC) analysis

A human melanoma tissue microarray (TMA) containing 62 cases of MM, 20 MM metastases and 18 benign nevus tissue cores was purchased from US Biomax, Rockville, USA. All donors were completely informed and gave their consent. Immunohistochemistry was performed as previously described with a rabbit monoclonal antibody against hnRNP K (conc. 1:250, 60 min at RT, Biozol, Eching, Germany) [41]. ZytoChem Plus AP Polymer-Kit was used for visualisation according to the manufacturer's instructions (Zytomed Systems, Berlin, Germany). The TMA cores were evaluated by light microscopy (Leica DM6000B, Leica, Wetzlar, Germany) and the *Diskus Mikroskopische Diskussion* image acquisition software (Carl Hilgers, Königswinter, Germany). IHC scores were obtained by assessing the intensity of nuclear and cytoplasmic staining for hnRNP K independently by two investigators (KS, SE), using the following scoring method: negative = 0, weak = 1, moderate = 2, strong = 3. IHC scores are presented as mean ± SD.

### Cell culture and cell transfection experiments

IPC-298 cells are originally derived from the primary cutaneous melanoma of a 64-year-old female patient. Histologically, the tumour has been classified as superficial spreading melanoma. Epitheliodendritic IPC-298 cells have been shown to exhibit cytoplasmic granular pre-melanosomes as well as mature melanosomes and carry a *NRAS* p.Q61L mutation [42, 43]. Cells were cultivated under standard cell culture conditions (37°C, 5%CO_2_) in RPMI 1640 medium (Gibco, Eggenstein, Germany) supplemented with 10% fetal calf serum (Boehringer Mannheim, Mannheim, Germany). Cells were sub-cultivated upon reaching 70% confluence and grown as a monolayer in plastic culture flasks (75 cm^2^) or dishes (25 cm^2^).

Transfection experiments were performed using the Lipofectamine 2000 transfection reagent (Invitrogen, Mannheim, Germany) and Silencer® Select hnRNP K siRNA (sequence: 3′-AUAAUCAUAGGUUUCAUCGta; 5´-CGAUGAAACCUAUGAUUAUtt) and Silencer® Select negative control siRNA #1 (both from Life Technologies, Waltham, USA) according to the manufacturer's instructions. After 48 h, transfected cells were harvested or treated according to the underlying experimental protocol. In the case of further treatment, the medium containing transfection reagents was replaced by standard cell culture medium after 48 h.

### Cell fractionation and western blot analysis

In brief, generation of cytoplasmic and nuclear fractionation was achieved by lysing cell pellets with lysis buffer (10 mM NaCl, 10 mM Tris-HCl, pH 7.6), followed by centrifugation for 10 minutes at 6000 g (4°C). The pellet was washed twice with low salt buffer (10 mM NaCl, 10 mM Tris-HCl, pH 7.6), resuspended in high salt buffer (500 mM NaCl, 10 mM Tris-HCl, 1% Triton X-100, pH 7.6) and incubated for 30 minutes on a rotating wheel (4°C). After lysing nuclei by 10 passages through an 18 G needle and centrifugation for 10 minutes at 13.000 g (4°C), the nuclear fraction was represented by the obtained supernatant. Immunoblotting was done according to standard methods. Before electrophoresis, protein concentrations were determined using the BCA Protein Assay Kit according to the manufacturer's instructions (Cell signaling technologies, Danvers, USA). Table 2 shows the antibodies and concentrations used in the study; β-Actin was used as loading control. Semi-quantitative gel analysis was performed using ImageJ software (V.1.46r, NIH, Bethesda, USA).

**Table 2:** List of antibodies used for western blotting

Antibody	Supplier	Dilution
**Primary antibodies**		
Rabbit anti-hnRNP K	Biozol	1:1000
Rabbit anti-phospho-ATM (Ser 1981)	Cell Signaling	1:1000
Mouse anti- β-Actin	Thermo Fisher	1:10000
Rabbit anti-GAPDH	Cell Signaling	1:10000
Rabbit anti-Histone 3	Cell Signaling	1:1000
**Secondary antibodies**		
Goat anti-rabbit (HRP-conjugated)	Thermo Fisher	1:10000
Rabbit anti-mouse (HRP-conjugated)	Thermo Fisher	1:10000

### *In vitro* X-ray irradiation

Irradiation of IPC-298 cells was performed by applying 240kV X-rays (YXLON Maxishot, Hamburg, Germany) using a 3 mm beryllium filter. The dose rate amounted to 17 mGy/sec at 13 mA. Absorbed dose was determined by a PTW Unidose dosimeter (PTW Freiburg GmbH, Freiburg, Germany). Control cells were stored under identical conditions at room temperature during irradiation experiments [27].

### NRAS mutation analysis

*NRAS* mutation status of IPC-298 cells was determined by DNA pyrosequencing on the PyroMark Q24 System (Qiagen, Hilden, Germany) following the manufacturer's instructions. After lysing cells in standard lysis buffer, creation of single-stranded DNA was conducted using the PyroMark Q24 Vacuum Workstation; the therascreen NRAS Pyro Kit (Qiagen, Hilden, Germany) was used to perform pyrosequencing according to the manufacturer's protocol. Sequence data was analyzed using the PyroMark Q24 Software (v. 2.0, Qiagen, Hilden, Germany).

### Immunofluorescence microscopy and detection of γH2AX/53BP1-Foci

IPC-298 cells were cultured in chamber slides (Thermo Fisher, Karlsruhe, Germany) as monolayer up to 70% confluence, treated according to the respective experimental protocol and fixed with ice-cold, phosphate-buffered aqueous formaldehyde solution (4%, Roti-Histofix®, Roth, Germany). Cells were permeabilized in 0.2% Triton-X 100 in PBS puffer and blocked for 30 min with 5% FCS in PBS before incubation with primary/secondary antibodies for 1 h at RT. Slides were mounted with VECTASHIELD® Mounting Medium containing 4, 6-diamidino-2-phenylindole (DAPI) for counterstaining of cell nuclei (Vector Laboratories, Burlingame, USA). Staining of actin filaments was performed with TexasRed-conjugated Phalloidin (conc. 1:40, Invitrogen, Mannheim, Germany). Primary antibodies: anti-hnRNP K (rabbit monoclonal, conc. 1:250, Biozol, Eching, Germany), γH2AX (mouse monoclonal, conc. 1:250, Millipore, Schwalbach, Germany) and 53BP1 (rabbit monoclonal, conc. 1:500, Acris, Herford, Germany). Fluorescence-labeled secondary antibodies: Alexa Fluor® 555-conjugate (goat polyclonal anti-rabbit, conc. 1:500) and Alexa Fluor® 488-conjugate (goat polyclonal anti-mouse, conc. 1:500, both from Life Technologies, Waltham, USA). Image acquisition was obtained by using a Zeiss Axioimager 2i fluorescence microscope (Carl Zeiss, Jena, Germany) and the ISIS fluorescence imaging system (MetaSystems, Altlussheim, Germany) [27]. For detection and quantification of DNA repair foci, only simultaneous γH2AX/53BP1 staining signals were counted and included into analysis by manual focus counting (n > 100).

### Clonogenic survival assay

IPC-298 cells (*n* = 100 per well, *n* = 200/well preliminary to irradiation) were seeded in 6 well plates and incubated for 24 h. Subsequent experiments were conducted according to the particular experimental protocol. After 9 days, colonies were stained with gentian violet and counted (> 50 cells per colony/> 10 cells per colony for transfection experiments). All experiments were performed in quadruplicate.

### Flow cytometry

Flow cytometry was performed using the BD FACSCalibur system (Fluorescence-activated cell sorter flow cytometer, BD, Heidelberg, Germany). Running samples with BD CellQuest Pro software (both from Becton, Dickinson and Company, New Jersey, USA), the number of events counted by the flow cytometer were at least 10000 per sample. All experiments were performed in triplicate. Co-staining for Annexin V and propidium iodide, respectively, was conducted using the Annexin-V-FLUOS Staining Kit (Roche Diagnostics, Basel, Switzerland), according to the manufacturer's instructions. Annexin V-positive and propidium iodide-negative cells were regarded as early apoptotic cells, while cells displaying Annexin V/propidium iodide co-staining represented the late apoptosis/necrotic cell fraction, respectively. Data analysis was done by performing quadrant statistics using BD CellQuest Pro software (BD, Heidelberg, Germany).

### Statistics

For the quantification of differences in nuclear respective cytoplasmic IHC scores between benign nevi, MM and MM metastases, we performed Kruskal-Wallis-test followed by Dunn's multiple comparison post-test. Differences in γH2AX/53BP1-foci repair at particular points of time upon IR and flow cytometry experiments were tested for significance performing ANOVA (Kruskal-Wallis-test) followed by Dunn's multiple comparison post-test or the Holm-Sidak method, where appropriate, using SigmaPlot software (v. 12.0, Systat Software, Erkrath, Germany). Plating efficiency (PE) and surviving fraction (SF) for clonogenic survival assays were calculated as follows: PE = (Colonies counted)/(Cells seeded per well)*100; SF = (Colonies counted)/((cells seeded per well) (PE/100)). Mean SFs of untreated control groups were set as reference basic value (100%). *P*-values of < 0.05 were regarded as statistically significant. Graph bars represent mean values ± standard deviation.

## Abbreviations

ATM: Ataxia telangiectasia mutated
BRAF: V-Raf murine sarcoma viral oncogene homolog B1
DDR: DNA damage response
DMSO: Dimethyl sulfoxide
DSB: Double strand break
ERK: Extracellular-signal-related kinase
FCS: Fetal calf serum
FITC: Fluorescein isothiocyanate
hnRNP K: Heterogeneous nuclear ribonucleoprotein K
IR: Ionizing radiation
IHC: Immunohistochemistry
MAPK: Mitogen-activated protein kinase
MEK: Mitogen/extracellular signal-related kinase
MDM-2: Mouse double minute-2 homolog
MM: Malignant melanoma
NRAS: Neuroblastoma rat sarcoma
PARP: Poly-ADP ribose polymerase
PI: Propidium iodide
PS: Phosphatidylserine
TMA: Tissue microarray
